# Supplementary material for: A chromosome-level genome assembly of the classical model nematode Panagrellus redivivus for comparative studies of parasitism evolution
Source: GigaByte. 2026 Oct 30;2026:gigabyte198. doi: 10.46471/gigabyte.198 (PMC13748681; doi:10.46471/gigabyte.198)
Supplement: Peer review history

# Reviewer report: Review MS


Reviewer name and names of any other individual's who aided in reviewer Oleksandr HolovachovDo you understand and agree to our policy of having open and named reviews, and having your review included with the published papers. (If no, please inform the editor that you cannot review this manuscript.)YesIs the language of sufficient quality?YesPlease add additional comments on language quality to clarify if needed
Are all data available and do they match the descriptions in the paper? NoAdditional CommentsDDBJ Sequence Read Archive gives 502 Bad Gateway when trying to use search function, so without direct links to accessions I can not check if the data is actually available.Are the data and metadata consistent with relevant minimum information or reporting standards? See GigaDB checklists for examples <a href="http://gigadb.org/site/guide" target="_blank">http://gigadb.org/site/guide</a>YesAdditional CommentsThe http://gigadb.org/site/guide page does not exist which means I can not properly answer this question. I assume the authors did their best to comply with the standards.Is the data acquisition clear, complete and methodologically sound?NoAdditional CommentsThere is no information if the contaminants were removed from the genome assembly, which makes it difficult to evaluate the correctness of assembly metrics and interpretations.Is there sufficient detail in the methods and data-processing steps to allow reproduction?YesAdditional CommentsIs there sufficient data validation and statistical analyses of data quality? NoAdditional CommentsIs there a specific reason that BlobToolKit was not used to validate the data? Blob plot and Snail plot would have been very useful and informative.Is the validation suitable for this type of data?YesAdditional CommentsWhich exact reference database was used in BUSCO comparison? Metazoa or Nematoda or Chromadorea? odb10 or odb12 or odb 12.1?Is there sufficient information for others to reuse this dataset or integrate it with other data?YesAdditional CommentsAny Additional Overall Comments to the AuthorRecommendationMinor Revision


# Reviewer report: Review MS


Reviewer name and names of any other individual's who aided in reviewer xiaobo wangDo you understand and agree to our policy of having open and named reviews, and having your review included with the published papers. (If no, please inform the editor that you cannot review this manuscript.)YesIs the language of sufficient quality?YesPlease add additional comments on language quality to clarify if needed
Are all data available and do they match the descriptions in the paper? NoAdditional Comments1. Although I was unable to access the DDBJ database, the SRA repositories of DDBJ and NCBI are fully interoperable and share identical dataset records. When I retrieved the two datasets with accession numbers DRR1089823 and DRR1089824 on the NCBI SRA platform, DRR1089823 is explicitly annotated as Illumina sequencing data, while DRR1089824 is annotated as Nanopore sequencing data, which is exactly opposite to the description of the sequencing platforms for these two datasets in the main text. Please carefully check and correct the relevant description in the manuscript to avoid misinterpretation of the experimental methodology by readers. 2. As confirmed by the official record at https://www.ncbi.nlm.nih.gov/sra/DRR1089823, the actual downloadable data size of the Illumina dataset (accession number DRR1089823) is only 585 Mb, which is more than two orders of magnitude smaller than the 72 Gb described in the main text.Are the data and metadata consistent with relevant minimum information or reporting standards? See GigaDB checklists for examples <a href="http://gigadb.org/site/guide" target="_blank">http://gigadb.org/site/guide</a>YesAdditional CommentsIs the data acquisition clear, complete and methodologically sound?YesAdditional CommentsIs there sufficient detail in the methods and data-processing steps to allow reproduction?NoAdditional CommentsPlease provide detailed protein homology information in gene annotation method.Is there sufficient data validation and statistical analyses of data quality? YesAdditional CommentsIs the validation suitable for this type of data?YesAdditional CommentsIs there sufficient information for others to reuse this dataset or integrate it with other data?YesAdditional CommentsAny Additional Overall Comments to the Author1. The total number of protein-coding genes annotated in this study differs by 6410 from the count reported in prior published genomes of the same or closely related species. This magnitude of difference is non-negligible and requires rigorous justification. A plausible explanation is that fragmentation of the previously reported genome assemblies led to gene breakage during annotation, where intact single genes were split into multiple fragmented or partial gene models, resulting in an inflated gene count in earlier studies. To rule out annotation bias and verify the completeness of the gene annotation in this work, the authors should perform BUSCO assessment of their annotation results. 2. The Fig2A legend states that “The estimated genome size was 85.2 Mb, with 0.46% heterozygosity and a k-mer coverage of 9.85×”, but these values are inconsistent with the k-mer frequency distribution, heterozygosity peak position, and coverage peak shown in the figure.RecommendationMajor Revision


# Reviewer report: Re-Review MS


Indicate in the comments box below whether you are happy with the changes made or if the manuscript is unacceptable.Comments on revised manuscriptThe authors has addressed most of my concerns. One more suggestion: Specify the accession numbers for the additional ~5.2 Gb of short-read data (besides the newly sequenced 585Mb data) in Data availability so readers can assess the total dataset composition.Indicate in the comments box below whether you are happy with the changes made or if the manuscript is unacceptable.Comments on revised manuscriptThe authors has addressed most of my concerns. One more suggestion: Specify the accession numbers for the additional ~5.2 Gb of short-read data (besides the newly sequenced 585Mb data) in Data availability so readers can assess the total dataset composition.


# Editor report: Accept


Editor’s AssessmentThe manuscript is ready for formal acceptance.Editor’s AssessmentThe manuscript is ready for formal acceptance.
